# A mobilization poster stimulates early in-hospital rehabilitation after cardiac surgery: a prospective sequential-group study

**DOI:** 10.1186/s13019-023-02173-w

**Published:** 2023-03-09

**Authors:** Frank R. Halfwerk, Nicole Wielens, Stephanie Hulskotte, Marjolein Brusse-Keizer, Jan G. Grandjean

**Affiliations:** 1 Department of Cardio-Thoracic Surgery, Thorax Centrum Twente, Medisch Spectrum Twente, PO Box 50000, 7500 KA Enschede, The Netherlands; 2 Department of Biomechanical Engineering, TechMed Centre, University of Twente, PO Box 217, 7500 AE Enschede, The Netherlands; 3 Medical School, Medisch Spectrum Twente, PO Box 50 000, 7500 KA Enschede, The Netherlands

**Keywords:** Cardiac rehabilitation, Early ambulation, Exercise, Physical therapy, Thoracic surgery

## Abstract

**Background:**

Patients infrequently mobilize at the surgical ward after cardiac surgery. Inactivity results in prolonged hospital stay, readmissions and increased cardiovascular mortality. Next, the course of in-hospital mobilization activities for patients is unclear. The aim was to evaluate early mobilization after heart surgery with a mobilization poster on the Activity Classification Guide for Inpatient Activities score from the American College for Sports Medicine (ACSM). Second, to develop a Thorax Centrum Twente (TCT) score to assess distinctive activities performed.

**Methods:**

A poster was developed for the Moving is Improving! study to stimulate hospital mobilization after heart surgery. In this sequential-group study at a cardiothoracic surgery ward, 32 patients were included in the usual care group and 209 patients in the poster mobilization group. Change of ACSM and TCT scores over time were both defined as primary endpoints. Secondary endpoints included length of stay and survival. A subgroup analysis for coronary artery bypass grafting (CABG) was performed.

**Results:**

ACSM score increased during hospital stay (*p* < 0.001). No significant increase of ACSM score was observed with a mobilization poster (*p* = 0.27), nor in the CABG subgroup (*p* = 0.15). The poster increased mobility to chair, toilet, corridor (all *p* < 0.01) and cycle ergometer (*p* = 0.02) as measured by the activity-specific TCT scores, without differences in length of stay or survival.

**Conclusions:**

ACSM score measured day-to-day functional changes, without significant differences between the poster mobilization and usual care group. Actual activities measured with the TCT score did improve. The mobilization poster is now new standard care, and effects in other centers and other departments should be assessed.

***Trial registration*:**

This study does not fall under the ICMJE trial definition and was not registered.

**Supplementary Information:**

The online version contains supplementary material available at 10.1186/s13019-023-02173-w.

## Background

Elective cardio-thoracic surgery patients have an average hospital stay for 6 to 7 days after surgery in U.S.A. and Europe [1, 2], and an average postoperative stay of 9 days in China [3]. Patients often do not know why physical activity and mobilization is important, and stay in bed during hospital stay [4]. This leads to muscle loss and reduced aerobic capacity [5], prolonged hospital stay [6], and readmissions [7]. Hospitalized cardio-thoracic surgery patients lie in bed for the majority of the day [8], despite enhanced recovery after surgery protocols [9]. Rarely patients have their meal outside of bed or walked during hospital admission [10].

Guidelines do describe features of “phase I rehabilitation”, “inpatient” or “early mobilization”, e.g. postoperative mobilization [11, 12], yet focus mainly on outpatient rehabilitation. Almost 20% of postoperative patients do not receive inpatient cardiac rehabilitation, and when utilized less than 30% of the hospital days are covered [13], often without weekend day services [14].

In-hospital exercises include breathing techniques, transfer between bed and chair and walking along the ward [8]. This leads to less pain and depression as well as decreased length of stay [15], and these outcomes are associated with lower mortality, morbidity, and costs [16]. Early mobilization strategies increased the exercise capacity (6-min walking assessment distance from 377 to 444 m) [17], but did not meet the minimal clinically important difference [18].

Success of mobilization can be assessed with wearable devices [8], and patient-reported or professional scores e.g. the Activity Classification Guide for Inpatient Activities from the American College for Sports Medicine (ACSM) [19]. The latter is a 6-point score that describes activity classes of patients from sitting up in bed with assistance (class I) to doing self-care activities in the bathroom (class III) to independent frequent ambulation (class VI), see Table 1.

**Table 1:** Activity Classification Guide for Inpatient Activities (ACSM score)

Activity class I	Activity class II	Activity class III
• Sits up in bed with assistance • Does own self-care activities-seated, or may need assistance • Stands at bedside with assistance • Sits up in chair 15–30 min, 2–3 times per day	• Sits up in bed independently • Stands independently • Does self-care activities in bathroom-seated • Walks in room and to bathroom (may need assistance)	• Sits and stands independently • Does own self-care activities in bathroom, seated or standing • Walks in halls with assistance short distance (15-30 m)^a^ as tolerated, up to 3 times per day

Activity class IV	Activity class V	Activity class VI
Does own self-care and bathes • Walks in hall short distances (45-60 m)^b^ with minimal assistance, 3–4 times per day	• Walks in halls independently, moderate distances (75-150 m)^c^, 3–4 times per day	Independent ambulation on unit, 3 to 6 times per day

Table adapted from ACSM’s Guidelines for exercise testing and prescription, 6th edition [19]

Original text: ^a^50–100 ft; ^b^150 to 200ft; ^c^250 to 500ft

However, sensitivity of the ACSM score to measure day-to-day changes for cardio-thoracic surgery patients is unclear. Since the ACSM score aggregates activities in a composite score, it is unclear which individual activities patients perform during early mobilization. A new Thorax Centrum Twente (TCT) score focusing on actual activities might overcome this limitation.

Thus, the aim of the Moving is Improving! study is to evaluate the stimulating effect of early rehabilitation after heart surgery with a mobilization poster on functional independence measured with the ACSM and TCT score. The effects are compared to a usual care group. The hypotheses are that a mobilization poster improves in-hospital mobilization compared to a usual care group (UCG), and that the TCT score is able to estimate distinctive patient mobilization activities throughout hospital stay.

## Methods

This study is reported as per the STROBE recommendations on the quality of reporting observational studies [20]. The data presented in this study will be published openly available in 4TU.ResearchData data repository.

### Study design and study population

This single center, prospective observational sequential-group study was conducted at Thorax Centrum Twente (Medisch Spectrum Twente, Enschede, The Netherlands), a tertiary non-academic teaching hospital. Consecutive adult patients undergoing non-salvage cardiac surgery were included. Patients were excluded with a Katz Index of Independence in Activities of Daily Living ≤ 2 before surgery (i.e. all patients included were preoperatively independent in daily life mobilization) [21] and patients with an intensive care unit (ICU) stay longer than 72 h were also excluded from analysis.

**Fig. 1:**
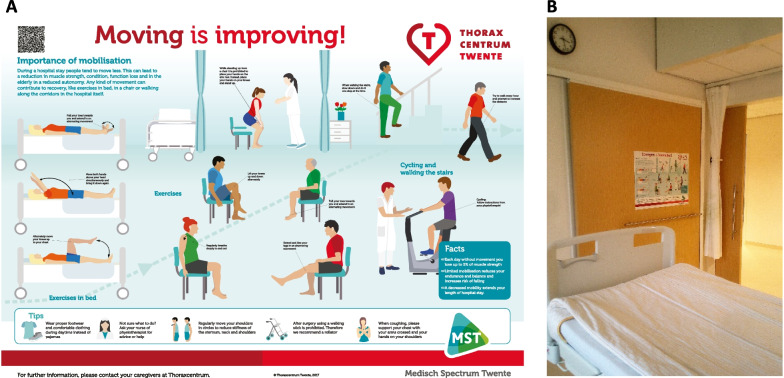
**A** Design of mobilization poster to promote early mobilization at cardio-thoracic surgery ward attached to every patient room; **B** Poster situated in patient room in original language (Dutch)

All patients were admitted to the ICU after surgery. An A1 paper size (84 × 59 cm) mobilization poster for each patient room was developed (Fig. 1) based on preliminary external work with a smaller A4 paper size leaflet [22].

The “Moving is Improving!” practice improvement initiative recruited from 03 to 20 October 2016 as UCG, and from 31 October 2016 to 22 November 2016 for the poster mobilization group (PMG). This practice improvement was initiated when nurses and physiotherapists observed that patients were not motivated for early mobilization. A best practice unit leadership program was started with the underlying study.

7 dedicated physical therapists trained for cardio-thoracic physiotherapy practice participated in the study. Physical therapists were trained in ACSM and TCT classification and a pocket card was handed out for daily use. Nurses and surgical staff were also educated on the importance of early mobilization. One physical therapy intern was added to the team in the PMG, and received similar training. A physiotherapist noted down patient-reported ACSM score daily at each patient room, and was collected after discharge. After interim analysis, the mobilization poster (Translated from Dutch to English, Fig. 1) was implemented as new standard care in the cardio-thoracic surgery ward and patients were also included from 10 September 2017 to 26 March 2018 (PMG).

ACSM score (see Table 1 for definitions) was used to compare UCG to PMG during postoperative hospital stay. No other changes than the poster were implemented during the study.

Change in in-hospital ACSM score and a more detailed Thorax Centrum Twente score (TCT) were defined as primary endpoints. Secondary endpoints included ICU length of stay, surgical ward stay and 30-, 120-day and overall survival. Follow-up on mortality was 100% and ended 1 February 2021. Baseline characteristics were determined based on EuroSCORE II definitions [23]. Rethoracotomy within 30 days, red blood cell transfusions, and rhythm problems were defined according to Netherlands Heart Registry definitions [24]. Temporary pacemaker leads were removed at postoperative day 2 to 5, depending on the type of surgery and underlying rhythm. Having a temporary pacemaker lead was no constraint for mobilization.

A 3 weeks interval of cardio-thoracic surgery determined UCG study size. A consecutive 3 weeks interval determined PMG size and was followed by 6 months use of the poster as new standard care (PMG).

The investigation conforms with the principles outlined in the Declaration of Helsinki [25]. This study was exempted from the Medical Research Involving Human Subjects Act by the Medical Ethics Committee Twente (METC Twente: K16-85) and was approved by the local institutional review board. Patients therefore did not sign informed consent.

### Development of TCT score

Six functional activities were taken from the ACSM score [19] and Functional Independence Measure dimensions [26]: lying in bed, sitting in a chair, walking to the toilet in the patients’ own hospital room, walking along the ward corridor, cycling at a cycle ergometer (stationary exercise bike) and walking the stairs. Four frequency descriptors were added: never, sometimes, often and always. A matrix of functional activities and frequency descriptors is provided in Additional file 1: File S1. A patient questionnaire for poster experience was sent by mail to patients after discharge (Additional file 1: File S2). In terms of validity, the TCT score was evaluated by calculating two-way mixed, consistency, average-measures intra-class correlation coefficients (ICC) to assess consistency between the ACSM and TCT scores. The inter-rater reliability was qualified as poor for ICC values less than 0.40, fair for values between 0.40 and 0.59, good for values between 0.60 and 0.74, and excellent for values between 0.75 and 1.0 [27].

### Exercise program and milestones

Patients received passive mobilization strategies, and potentially sitting on the edge of their bed or chair mobilization starting from the first postoperative day at ICU. Patients received physical therapy twice a day until the 3^rd^ postoperative day and then once a day in both groups, as is standard of care [8]. A standardized program starting at ICU discharge included:

- Day 1: Breathing exercises, coughing techniques, control mobilization upper- and lower extremities, transfer from bed to chair with assistance;
- Day 2: Exercises as on day 1. Self-transfer from bed to chair with or without assistance, ambulation with assistance for 20 m at surgical ward;
- Day 3: Exercises as on day 2. Ambulation with increase in distance (± 15 m) and frequency (3 times), cycling for 5–10 min with 0–10 Watt depending on hemodynamic stability;
- Day 4: Exercises as on day 3. Walking stairs (1 floor) with assistance, information on home mobilization, increase cycling duration (5–10 min) and power (10–15 Watt).

Each patient specific exercise program was based on evaluation findings, comorbidities and patient goals. The poster was discussed during physical therapy sessions, where exercises were shown. Patients were encouraged to continue mobilization activities as practiced with the physiotherapist. Furthermore, patients in the poster group were stimulated to practice the poster exercises regularly.

### Statistical analysis

Statistical analysis was performed with SPSS 28.0 (SPSS Inc, Chicago, IL). Results were considered statistically significant at the 5% level. Continuous variables were presented as mean with standard deviation (SD) or median with interquartile range (IQR) depending on the distribution. Continuous variables were tested for normality with visual inspection of histograms and skewness and kurtosis measures. Categorical variables were presented as number with corresponding percentages and compared between groups using a Fisher Exact Test. A Kaplan Meier curve with log rank test was used to test for differences in survival between UCG and PMG.

A linear mixed model was used to determine differences in ACSM and TCT scores between groups (UCG and PMG), differences over time (day-to-day scores) and interaction between group and time to study differences in the course of these scores over time between the two groups.

A sex subgroup analysis as encouraged by the Institute of Medicine [28] was performed. Furthermore a subgroup analysis of coronary artery bypass grafting (CABG) patients was planned for all endpoints because of different Dutch guidelines for CABG and non-CABG in-hospital mobilization [29].

## Results

**Fig. 2:**
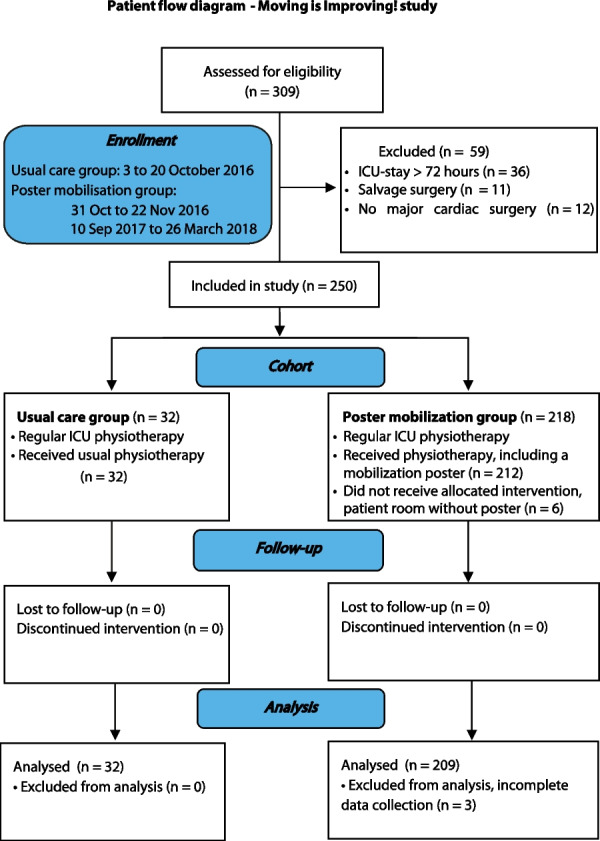
Patient Flow diagram of Moving is Improving! Study, based on CONSORT [30]. First the UCG cohort was completed, after which PMG cohort followed. Six patients were in a patient room without a poster and did not receive the poster. Three patients from PMG were excluded because of incomplete data collection. Thus, 241 patients were included for analysis with 32 patients in the UCG cohort and 209 patients in the PMG cohort. ICU = Intensive care unit

Out of 309 patients selected for eligibility, with 37 patients in the UCG (UCG) and 272 patients in the poster group (PMG), in total 59 patients were excluded based on prolonged ICU stay over 72 h (n = 36), salvage surgery (n = 11) and absence of major cardiac surgery (n = 12) (Fig. 2).

### Baseline characteristics

Patients had a mean age of 67 years, a BMI of 28 kg/m^2^, over 50% had multivessel disease and 16% a recent myocardial infarction. There were no significant differences between groups for baseline or in-hospital characteristics (Table 2). Median ICU stay was 1.5 [1, 2] day in UCG and 1 [1, 2] day in PMG (*p* = 0.92) with a median surgical ward stay of 4 days in both groups (UCG 4 [2.5–4] and PMG 4 [3–5], *p* = 0.51, Table 3).

**Table 2:** Baseline and procedural characteristics

Variable	Overall (n = 241)	UCG (n = 32)	PMG (n = 209)	*P* value
*Baseline characteristics*				
Age, years	67 ± 11	66 ± 12	68 ± 11	0.32
Sex, male	175 (73%)	24 (75%)	151 (72%)	0.83
Body Mass Index, kg/m^2^	28 ± 4.2	29 ± 4.5	28 ± 4.1	0.20
Diabetes	66 (27%)	9 (28%)	57 (27%)	0.94
Multivessel disease	138 (69%)	17 (77%)	121 (68%)	0.37
Recent myocardial infarction	39 (16%)	6 (19%)	33 (16%)	0.67
*Left ventricular function*				0.13
Poor, < 30%	8 (3.3%)	2 (6.3%)	6 (2.9%)	
Moderate, 30–50%	43 (18%)	2 (6.3%)	41 (20%)	
Good, > 50%	190 (79%)	28 (88%)	162 (78%)	
COPD	26 (11%)	2 (6.2%)	24 (12%)	0.55
Extracardiac arteriopathy	28 (12%)	1 (3.1%)	27 (13%)	0.14
Neurological dysfunction	6 (2.5%)	0 (0%)	6 (2.9%)	0.99
Previous cardiac surgery	11 (4.6%)	0 (0%)	11 (5.3%)	0.37
*NYHA class*				0.13
I	171 (71%)	28 (88%)	143 (69%)	
II	49 (20%)	4 (13%)	45 (22%)	
III	18 (7.5%)	0 (0%)	18 (8.7%)	
IV	2 (0.8%)	0 (0%)	2 (1.0%)	
*Urgency*				0.53
Elective	150 (62%)	19 (59%)	131 (63%)	
Urgent	85 (35%)	13 (41%)	72 (34%)	
Emergency	6 (2.5%)	0 (0%)	6 (2.9%)	
Salvage	0 (0%)	0 (0%)	0 (0%)	
EuroSCORE I, logistic	3.7 [1.9 – 7.4]	2.8 [1.7 – 5.8]	3.7 [1.9 – 7.5]	0.18
EuroSCORE II	1.3 [0.92 – 2.3]	1.1 [ 0.83 – 1.7]	1.4 [ 0.93 – 2.3]	0.16
*Periprocedural characteristics*				
Type of surgery				0.29
CABG	142 (59%)	19 (59%)	123 (59%)	
Valve surgery	55 (23%)	5 (16%)	50 (24%)	
CABG + valve surgery	21 (8.7%)	2 (6.3%)	19 (9.1%)	
TAVI	12 (5.0%)	3 (9.4%)	9 (4.3%)	
Ascending aorta surgery	6 (2.5%)	2 (6.3%)	4 (1.9%)	
Other surgery	5 (2.1%)	1 (3.1%)	4 (1.9%)	
Cardiopulmonary bypass	162 (71%)	18 (62%)	144 (72%)	0.27
Cardiopulmonary bypass time, min	102 [79—128]	97 [62 – 137]	102 [80—127]	0.56

Data are means ± SD or Medians [IQR] or numbers (proportions)

*CABG* Coronary artery bypass grafting, *COPD* Chronic obstructive pulmonary disease, *NYHA* New York Health Association, *PMG* Poster mobilization group, *TAVI* Transcatheter aortic valve implantation, *UCG* Usual care group

**Table 3:** Postoperative characteristics and survival

Variable	Overall (n = 241)	UCG (n = 32)	PMG (n = 209)	*P* value
Intensive care unit stay, days	1 [1, 2]	1.5 [1, 2]	1 [1, 2]	0.92
Surgical ward stay, days	4 [3–5]	4 [2.5–4.5]	4 [3–5]	0.51
Rethoracotomy within 30 days	13 (6.4%)	0 (0%)	13 (7.1%)	0.75
Red blood cell transfusion	35 (15%)	4 (14%)	31 (16%)	0.99
Rhythm problems (atrial fibrillation and resuscitation)	56 (25%)	4 (14%)	52 (26%)	0.17
*Discharge to*				0.36
Home	154 (64%)	24 (75%)	130 (62%)	
Referring hospital	86 (36%)	8 (25%)	78 (37%)	
Other	1 (0.4%)	0 (0%)	1 (0.47%)	
*Survival*				
30-Day survival	241 (100%)	32 (100%)	209 (100%)	0.99
120-Day survival	240 (99.6%)	32 (100%)	208 (99.5%)	0.70
365-Day survival	237 (98%)	31 (97%)	206 (99%)	0.48

Data are Medians [IQR] or numbers (proportions)

*ACSM* Activity Classification Guide for Inpatient Activities score from the American College for Sports Medicine, *PMG* Poster mobilization group, *TCT* Thorax Centrum Twente score, *UCG* Usual care group

Median follow-up on mortality was 1574 days in UCG and 1187 days in PMG. There was no difference in mortality at all timepoints (Table 3, Additional file 1: File S3). No complications such as wound or sternum dehiscence or ventricular tachycardia related to early mobilization were reported.

### American College of Sports Medicine (ACSM) functional score

**Fig. 3:**
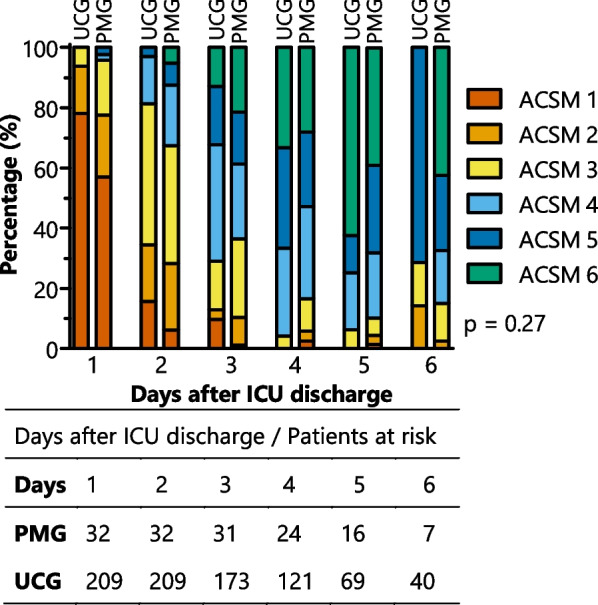
ACSM scores after intensive care unit discharge for the usual care group (UCG; left bars) and poster mobilization group (PMG; right bars). ACSM score changed significantly over time (*p* < 0.001), no difference between both groups was observed (*p* = 0.27)

Median ACSM score one day after ICU discharge was 1 [1] in UCG and 1 [1, 2] in PMG (day-to-day boxplot, see Additional file 1: File S4). Overall, ACSM scores increased significantly from postoperative admission to the surgical ward and discharge (*p* < 0.001), with a plateau phase starting from day 4 (Fig. 3). ACSM score was not significantly affected by the mobilization poster (*p* = 0.27), see Additional file 1: Tables S5.3 and S5.4.

### TCT descriptive score with actual activities

Incomplete data collection for one or multiple days resulted in exclusion from analysis for 28 patients. In total 32 UCG-patients and 188 PMG-patients were included in TCT score analysis. Overall, TCT scores increased significantly from postoperative admission to the surgical ward and discharge (*p* < 0.001).

**Fig. 4:**
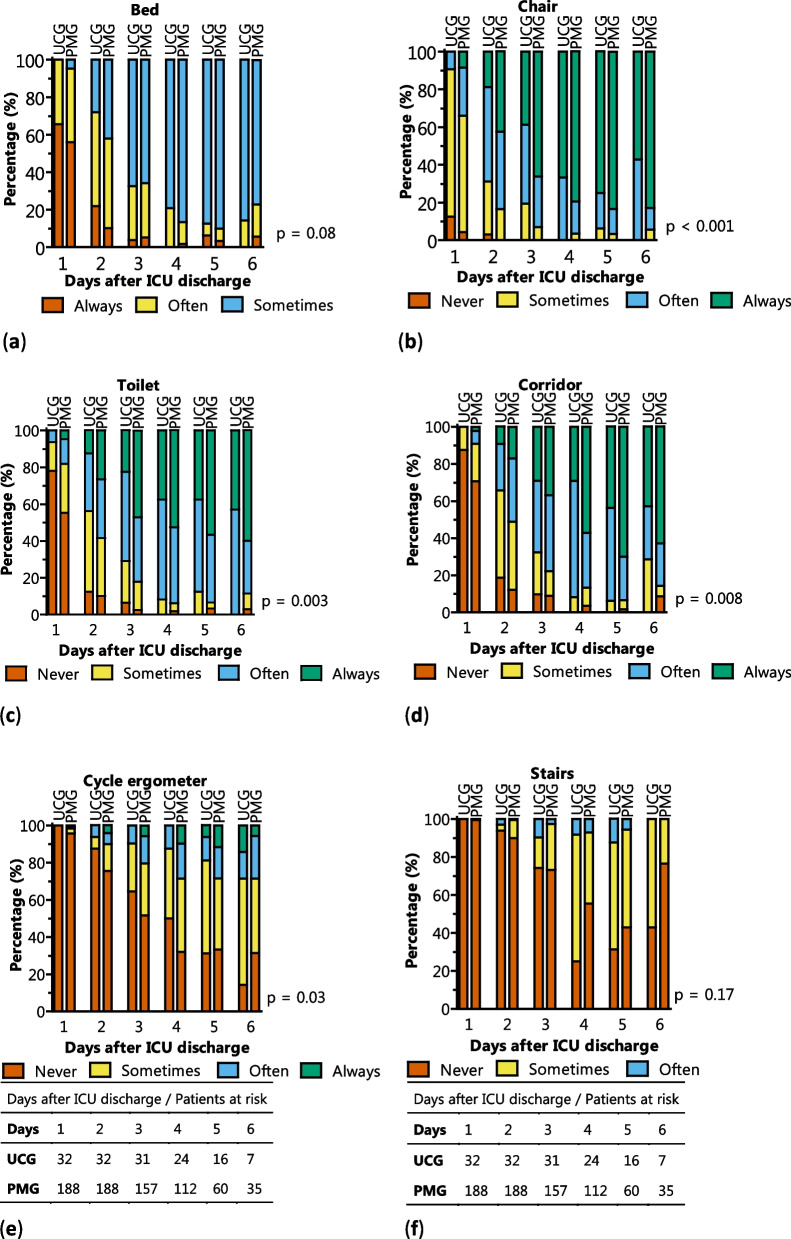
**a**–**f** TCT score development during hospital stay. Every individual TCT score, bed, chair, toilet, corridor, cycle ergometer and stairs was significantly different over time (*p* < 0.001). A significant improvement of scores chair, toilet, corridor and cycle ergometer was observed in the poster mobilization group (PMG) compared to the usual care group (UCG) as determined with mixed model analysis. Depicted *p* values are for group effect (UCG vs PMG)

Every individual TCT score, bed, chair, toilet, corridor, cycle ergometer and stairs was significantly different over time as determined in a linear mixed model (*p* < 0.001). TCT scores for chair, toilet, corridor and cycle ergometer were significantly higher in the PMG (see Fig. 4 and Additional file 1: Tables S5.3 and S5.4).

ACSM and TCT scores had a high degree of agreement, with ICCs in the good range for bed (0.73) and chair (0.74), excellent for toilet (0.75) and corridor (0.79), and poor for cycle ergometer (0.38) and stairs (0.21), see Additional file 1: File S6.

### Subgroup analysis for sex, and CABG

Men had an overall significant higher median ACSM score of 4 [2 to 5] throughout the stay compared to women, 3 [2 to 4] (*p* < 0.001). Furthermore, there was a significant difference in the course of the ACSM score over time between men and women (*p* < 0.001, Additional file 1: File S4). There was no difference in ACSM score for sex between UCG and PMG (*p* = 0.23). Differences in baseline characteristics and potential effects on ACSM score were assessed (Additional file 1: File S4). Differences in baseline characteristics such as age, NYHA class or EuroSCORE I and II did not improve the linear mixed model with sex. The difference in ACSM score between men and women was persistent in all models.

A subgroup analysis for CABG patients included 21 patients in UCG and 142 patients in PMG. There were no differences in baseline or periprocedural characteristics (see Additional file 1: Tables S5.1 and S5.2). ACSM was significantly different over time (*p* < 0.001). No significant effect of the mobilization poster was observed (*p* = 0.15) in the CABG subgroup (Additional file 1: Table S5.3). In the CABG subgroup TCT scores for bed, chair, toilet, corridor and cycle ergometer were significantly higher in the poster group (Additional file 1: Table S5.3).

## Discussion

The Moving is Improving! study evaluated the stimulating effect of a mobilization poster on early mobilization after heart surgery, as measured with the ACSM and TCT scores.

### Measuring ACSM functional score for cardiac surgery patients

ACSM score was sensitive to measure daily improvement of patient functional activities after heart surgery. A stabilization pattern was observed 4 to 5 days after ICU discharge. This is in line with accelerometer data obtained at our surgical ward [8]. The mobilization poster did not increase ACSM score in the overall group (*p* = 0.27) or CABG subgroup (*p* = 0.15).

We are the first to report results of an “aggressive phase I mobilization” strategy [31]. Our study shows that early mobilization is safe and results in increased activity levels without compromising safety. The early mobilization protocol is comparable in physical activity to other published protocols [32]. Afxonidis et al. intensified early postoperative physiotherapy sessions after cardiac surgery starting at the ICU, and reached milestones during hospital stay compared to our Day 2 protocol [15]. Physical therapists in Australia and New Zealand reported in a national survey that all patients are sitting out of bed at first postoperative day and 20% completed one flight of stairs on day 4 after uncomplicated CABG [14]. In our study, 88 to 96% sat on a chair at first postoperative day, 36 to 48% cycled at a cycle ergometer on day 3 and 45 to 75% walked the stairs on day 4 (see Additional file 1: File S7).

The ACSM score increased drastically in this study from day 1 to day 4. That means that implementation of this poster in more conservative mobilization programs focusing on lower metabolic equivalents of physical activity [15] are likely to observe an increase in ACSM score during hospital stay as well.

Higher ACSM scores were observed in men who might have a more competitive attitude to mobilization or overestimate self-reported activities. Self-reported patient activities were aligned with nursing records and with activities performed during physical therapy sessions. Most of these activities were unsupervised, and self-reported activities could therefore shift a borderline score towards a higher level. Also, women might be more hesitant to mobilize after surgery. This finding is in line with a study on postoperative mobilization after total hip arthroplasty [33]. No differences in in-hospital mobilization after cardiac surgery were observed between men and women in another study where accelerometers were used [8]. Further research should quantify this difference using objective data from activity trackers in a larger study. If the difference between men and women persists in future studies, physical therapists should develop motivational interviewing strategies to reduce women patients’ hesitance to mobilization.

In the Netherlands, receiving CABG is an absolute indication for cardiac rehabilitation, while valve surgery is a relative indication [29]. Therefore, a subgroup analysis for CABG was planned. No differences were found in CABG mobilization activities compared to the overall group, with a similar non-significant effect on ACSM score for the PMG (*p* = 0.15).

### TCT descriptive score

The mobilization poster had a significant impact on sitting in a chair, walking to the toilet and along the corridor and cycling at a cycle ergometer. No difference in TCT score for bed and stairs was found. This effect was persistent in the CABG subgroup. Patients generally only lay in bed at day 1 after ICU discharge and this did not change between UCG and PMG. Walking the stairs is a discharge target and thus did not change. These TCT scores might be removed in future studies to reduce administrative load for physical therapists.

Classification of patient mobility with the TCT scores had an overall high agreement with the ACSM score, with ICCs in the good to excellent range for bed, chair, toilet and corridor. There was poor agreement for cycle ergometer and stairs, which was no surprise because these activities are not described in the ACSM score at all. Last, TCT score was planned as an individual score per activity. Future studies might include a cumulative day score for comparison and for potentially clinical cut-off selection.

### Study limitations

Scoring was based on self-reported activities and was not blinded, potentially introducing bias. A study in our center used accelerometer measurements for objective qualification of activities, and found similar results [8].

Next, our prospective sequential-group study might include bias compared to a randomized controlled trial, and did not allow for extension of the control group, resulting in unequal group sizes. After the initial PMG group and extensive analysis, poster mobilization was continued as new standard. In this study, baseline characteristics were balanced between both groups (Table 2). Furthermore, a parallel randomized design would allow for informal cross-over as patients might see or discuss the poster at the surgical ward. Last, type I error might have been introduced with 7 primary endpoints (1 ACSM score and 6 TCT scores). With a Holm-Bonferroni correction for our primary endpoints, we still find significant results in our mixed model analysis (Additional file 1: Table S5.3) for chair (*p* = 0.007), toilet (*p* = 0.012), corridor (*p* = 0.025), and not cycle ergometer (*p* = 0.08).

A linear mixed model was used as length of stay differed between patients and repeated ACSM or TCT measures from the same patient are more similar than responses from other patients. A sample size of 32 patients in the control group might be too small to find a significant effect of a mobilization poster for a composite measure such as ACSM score. Contrarily, the TCT scores focusing on one activity only were able to find an improved effect of the mobilization poster in this study population.

ACSM and TCT scores sitting, and walking to the toilet and corridor were higher in the PMG at the first postoperative day. Preoperative ACSM and TCT scores were not collected the day before surgery. All patients were independent in daily life mobilization, as determined with the Katz Index of Independence in Activities of Daily Living [21]. It is unclear if the poster benefits patients as early as day one, or alternatively, that the patient groups differed already at ICU discharge. As there was no patient selection, and both cohorts were in a short time span, we do not expect preoperative differences and address this increase to be the poster effect.

### Future work

Other patient groups with intermediate to long hospital stay might benefit from early mobilization. At the cardiology ward and other wards early mobilization for patients with or without a mobilization poster should be evaluated using the ACSM or TCT score. Also congenital, heart transplant and heart assist device patients might benefit from a mobilization poster. Effects of prehabilitation for frail patients [34], patients with overweight or preoperatively expected long cardiopulmonary bypass times [35] for complex cardiac surgery can also be studied with these measures.

The poster group used an A1 paper size poster during ward stay. Functional activities might increase faster with a comprehensive approach including lifestyle changes [36], and using digital persuasive technology (telerehabilitation) focusing on the current activity level and milestones. Future work should focus on patient-specific information and exercises that match the current functional level of patients recovering from cardiac surgery based on self-reported or wearable device measurements [6, 8].

## Conclusion

ACSM functional score showed to be sensitive to measure day-to-day changes of patients at a surgical ward after cardiac surgery, yet no difference in effects between the mobilization poster and usual care group was observed. Higher ACSM scores were found for men. An early mobilization strategy using a mobilization poster however significantly increased sitting in a chair, walking to the toilet and corridor and cycling at a cycle ergometer as measured with the TCT score.

Implementation of a mobilization poster to promote a more active behavior after cardiothoracic surgery is a low-cost approach and should be implemented in other hospitals. Both the ACSM or TCT score can be used for evaluation of postoperative mobilization.

## Supplementary Information

**Additional file 1.** Supplementary tables and figures.

## Data Availability

The data presented in this study are openly available in 4TU.ResearchData after publication of this manuscript (10.4121/22001927). The mobilization poster (Fig. 1A) is available for reuse under an open CC-BY-NC-SA license with options for color tailoring.

## Abbreviations

ACSM: American College for Sports Medicine
BMI: Body mass index
CABG: Coronary artery bypass grafting
COPD: Chronic obstructive pulmonary disease
ICC: Intra-class correlation coefficient
ICU: Intensive care unit
NYHA: New York Health Association
PMG: Poster mobilization group
TAVI: Transcathether aortic valve implantation
TCT: Thorax Centrum Twente
UCG: Usual care group
