# Supplementary material for: A mobilization poster stimulates early in-hospital rehabilitation after cardiac surgery: a prospective sequential-group study
Source: J Cardiothorac Surg. 2023 Mar 9;18:83. doi: 10.1186/s13019-023-02173-w (PMC9999498; doi:10.1186/s13019-023-02173-w)
Supplement: Supplementary file 1 — Additional file 1. Supplementary tables and figures. [file 13019_2023_2173_MOESM1_ESM.docx]

**A Mobilization Poster stimulates Early In-Hospital Rehabilitation after Cardiac Surgery: A prospective sequential-group study**

**Frank R. Halfwerk ^1, 2 *^, Nicole Wielens ^1^, Stephanie Hulskotte ^1^, Marjolein Brusse-Keizer ^3^, Jan G. Grandjean ^1, 2^**

1 Thoraxcentrum Twente, Medisch Spectrum Twente, PO Box 50 000, 7500 KA Enschede, The Netherlands

2 Dept. of Biomechanical Engineering, TechMed Centre, University of Twente, PO Box 217, 7500 AE Enschede, The Netherlands

3 Medical School, Medisch Spectrum Twente, PO Box 50 000, 7500 KA Enschede, The Netherlands

**Address for correspondence:** Dr Frank R. Halfwerk, Dept. of Cardio-Thoracic Surgery, Thoraxcentrum Twente, Medisch Spectrum Twente, P.O. Box 50000, 7500 KA Enschede, The Netherlands, [f.halfwerk@mst.nl](mailto:f.halfwerk@mst.nl) , T: +31(0)631751406, F: +31(0)534876169, ORCiD: 0000-0003-2928-9728

**The additional files corresponding to this manuscript are:**

- File S1 Table, Thorax Centrum Twente score matrix (Dutch and English), and ACSM score
- File S2 Figure, Patient Questionnaire (English translated questionnaire)
- File S3 Figure, Kaplan-Meier curve with survival probability
- File S4 Figures and table, ACSM and sex day-to-day boxplot, sex subgroup characteristics
- File S5 Tables, Characteristics of CABG subgroup and effects on ACSM/TCT scores
- File S6 Tables, Validation of TCT and ACSM score and scores at POD1 and discharge
- File S7 Table, Milestone analysis of Moving is Improving! Study

***File S1 – TCT Matrix of functional activities and frequency descriptors***

| \| **Date** \| **Always/Mostly** \| **Often** \| **Sometimes / Rarely** \| **Never/N.A.** \| \| --- \| --- \| --- \| --- \| --- \| \| **Bed** \| Continuously in bed (1) \| Except during meals (2) \| Only after lunch / at night (3) \| N.A \| \| **Chair** \| Mainly in chair (4) \| 2-3 hours / day (3) \| Max 30 min / day (2) \| Never (1) \| \| **Toilet** \| Frequently independent (4) \| Independent 1-2x (3) \| Toilet chair / with supervision (2) \| Never / N.A. (1) \| \| **Corridor** \| > 5x per day (4) \| > 20m / max 4x per day (3) \| Max 20m / < 2x per day (2) \| Never / N.A. (1) \| \| **Home trainer** \| Independent, > 2x (4) \| Independent, 2x (3) \| With supervision, max 1x (2) \| Never / N.A. (1) \| \| **Stairs** \| N.A. \| Independent (3) \| With supervision (2) \| Never / N.A. (1) \|   **Translated Thorax Centrum Twente score form** |
| --- | --- | --- | --- | --- | --- | --- | --- | --- | --- | --- | --- | --- | --- | --- | --- | --- | --- | --- | --- | --- | --- | --- | --- | --- | --- | --- | --- | --- | --- | --- | --- | --- | --- | --- | --- |
| **Original Dutch Thorax Centrum Twente score form**   \| **Datum** \| **Vaak/vooral** \| **Regelmatig** \| **Soms / Zelden** \| **Nooit/n.v.t.** \| \| --- \| --- \| --- \| --- \| --- \| \| **Bed** \| Bedlegerig (1) \| Behalve tijdens eten (2) \| Rustuur / ’s nachts (3) \| n.v.t. \| \| **Stoel** \| Vooral op de stoel (4) \| 2-3 uur / dag (3) \| Max 30 min / dag (2) \| Niet (1) \| \| **Toilet** \| Frequent zelfstandig (4) \| Zelfstandig tot 2x (3) \| Po stoel / met begeleiding (2) \| Nooit / Nvt (1) \| \| **Gang** \| > 5x per dag (4) \| > 20m / max 4x per dag (3) \| Max 20m / < 2x per dag (2) \| Nooit / Nvt (1) \| \| **Fietsen** \| Zelfstandig, > 2x (4) \| Zelfstandig, 2x (3) \| Met begeleiding max 1x (2) \| Nooit / Nvt (1) \| \| **Trap** \| n.v.t. \| Zelfstandig (3) \| Met begeleiding (2) \| Nooit / Nvt (1) \| |

| **Table S1.** Activity Classification Guide for Inpatient Activities (ACSM score) | | |
| --- | --- | --- |
| **Activity class I** | **Activity class II** | **Activity class III** |
| • Sits up in bed with assistance  • Does own self-care activities-seated, or may need assistance  • Stands at bedside with assistance  • Sits up in chair 15-30 minutes, 2-3 times per day | • Sits up in bed independently  • Stands independently  • Does self-care activities in bathroom-seated  • Walks in room and to bathroom (may need assistance) | • Sits and stands independently  • Does own self-care activities in bathroom, seated or standing  • Walks in halls with assistance short distance (15-30m)^a^ as tolerated, up to 3 times per day |
| **Activity class IV** | **Activity class V** | **Activity class VI** |
| Does own self-care and bathes  • Walks in hall short distances (45-60m)^b^ with minimal assistance, 3-4 times per day | • Walks in halls independently, moderate distances (75-150m)^c^, 3 - 4 times per day | Independent ambulation on unit, 3 to 6 times per day |
| Table adapted from ACSM’s Guidelines for exercise testing and prescription, 7^th^ edition [1].  Original text: ^a^ 50-100 ft; ^b^ 150 to 200ft; ^c^ 250 to 500ft | | |

***File S2 - Patient Questionnaire (English and original Dutch questionnaire)***

A patient questionnaire for poster experience was developed with 13 rubric questions and one grading question. Both English and original Dutch questionnaire are attached.

Out of the 45 questionnaires that were sent by mail to patients from the first T_1_ group, 32 were returned with a respective response rate of 71%. Patients agreed (53%) or strongly agreed (31%) that the poster was clear (see Figure 5). Similar responses were obtained on clearness of the exercises (66% agreed and 28% strongly agreed). Only 19% of these patients (strongly) agreed that the poster needs additional explanation, while 56% (strongly) disagreed. No patient found the poster pushy.

| **** |
| --- |
| *Figure S2.* *Bar chart representation of patient questionnaire on mobilization poster experience* |

One patient did not reply questions on the backside of the questionnaire. By accident, a question bullet was missing for the “clearness of the poster” question and 15 patients did not respond to this question.

Response: 32/45 people from T­_1_-group (oct-nov 2016).

| Table S2.1. Responses from patients on mobilization poster experience | | | | | | | |
| --- | --- | --- | --- | --- | --- | --- | --- |
|  | Strongly disagree | Disagree | Neutral | Agree | Strongly Disagree | Don’t know | Missing |
| The poster |  |  |  |  |  |  |  |
| is clear | 0 | 3 | 2 | 17 | 10 | 0 | 0 |
| is understandable | 0 | 2 | 4 | 15 | 11 | 0 | 0 |
| helped me | 1 | 4 | 5 | 14 | 6 | 2 | 0 |
| needs additional explanation | 1 | 17 | 4 | 5 | 1 | 3 | 1‡ |
| is pushy | 4 | 22 | 4 | 0 | 0 | 1 | 1‡ |
| matches your recovery process | 1 | 2 | 5 | 18 | 5 | 1 | 0 |
| is motivating | 0 | 3 | 5 | 18 | 6 | 0 | 0 |
| gave me new information | 0 | 3 | 7 | 15 | 4 | 2 | 1‡ |
| is to my opinion, complete | 0 | 0 | 2 | 11 | 2 | 2 | 15† |
| The exercizes |  |  |  |  |  |  |  |
| are clear | 0 | 0 | 1 | 21 | 9 | 1 | 0‡ |
| are well executionable | 0 | 0 | 3 | 20 | 8 | 1 | 0‡ |
| I would like to |  |  |  |  |  |  |  |
| have seen the poster before surgery | 1 | 2 | 13 | 8 | 5 | 2 | 1‡ |
| receive the poster at discharge on A4-paper | 1 | 4 | 5 | 12 | 7 | 2 | 1‡ |
| † During data collection, it was noted that for this question, “bullets” were missing, resulting in many missing values | | | | | | | |
| ‡ One patient did not fill in the questions on the backside of the paper | | | | | | | |

Please give a grade for our intervention poster:

| Grade | 1 | 2 | 3 | 4 | 5 | 6 | 7 | 8 | 9 | 10 |
| --- | --- | --- | --- | --- | --- | --- | --- | --- | --- | --- |
| Number of responses | 0 | 1 | 0 | 0 | 0 | 1 | 5 | 21 | 1 | 1 |

For information converting grading systems in the Netherlands into U.S. or UK-systems, please see: <https://students.uu.nl/sites/default/files/geo-grading-systems-holland-vs-us-uk.pdf>

| Table S2.2. Original Dutch responses from patients on mobilization poster experience | | | | | | | |
| --- | --- | --- | --- | --- | --- | --- | --- |
|  | Volledig oneens | Oneens | Neutraal | Eens | Volledig eens | Weet niet | Ontbrekend |
| De poster is overzichtelijk | 0 | 3 | 2 | 17 | 10 | 0 | 0 |
| De poster is begrijpelijk | 0 | 2 | 4 | 15 | 11 | 0 | 0 |
| De poster heeft mij geholpen | 1 | 4 | 5 | 14 | 6 | 2 | 0 |
| De poster heeft aanvullende uitleg nodig | 1 | 17 | 4 | 5 | 1 | 3 | 1‡ |
| De poster is opdringerig | 4 | 22 | 4 | 0 | 0 | 1 | 1‡ |
| De poster sluit aan bij uw herstel | 1 | 2 | 5 | 18 | 5 | 1 | 0 |
| De poster werkt motiverend | 0 | 3 | 5 | 18 | 6 | 0 | 0 |
| De poster heeft mij nieuwe informatie gegeven | 0 | 3 | 7 | 15 | 4 | 2 | 1‡ |
| De oefeningen zijn duidelijk | 0 | 0 | 1 | 21 | 9 | 1 | 0‡ |
| De oefeningen zijn goed uitvoerbaar | 0 | 0 | 3 | 20 | 8 | 1 | 0‡ |
| Ik had de poster graag voor de operatie al gezien. | 1 | 2 | 13 | 8 | 5 | 2 | 1‡ |
| De poster is naar mijn idee volledig | 0 | 0 | 2 | 11 | 2 | 2 | 15† |
| Ik zou de poster graag bij ontslag krijgen op A4 formaat | 1 | 4 | 5 | 12 | 7 | 2 | 1‡ |

Geef een rapportcijfer voor de interventieposter:

| Rapportcijfer | 1 | 2 | 3 | 4 | 5 | 6 | 7 | 8 | 9 | 10 |
| --- | --- | --- | --- | --- | --- | --- | --- | --- | --- | --- |
| Aantal reacties | 0 | 1 | 0 | 0 | 0 | 1 | 5 | 21 | 1 | 1 |

***File S3 – Kaplan-Meier curve with survival probability***

|  |
| --- |
| **Figure S3.** Survival proportions of usual care group (UCG) and poster mobilization group (PMG) showing no significant difference in survival at all time points (p > 0.47) as determined with a log rank test. |

***File S4 – ACSM day to day boxplot and sex subgroup analysis***

|  |
| --- |

Figure S4a. ACSM development in time. No difference between usual care group and poster mobilization group was found (p = 0.27). Whiskers show the 25th and 75th percentile ± 1.5 times interquartile range (Tukey box-and-whiskers plot), outliers are depicted with dots.

Figure S4b. Sex differences in ACSM development. Men had a significantly higher ACSM score compared to women (p < 0.001)

***Tables S4 – Characteristics of sex subgroup and effects on ACSM score***

| **Table S4.1.** Baseline characteristics of sex subgroup patients | | | |
| --- | --- | --- | --- |
| **Variable** | **Male (n = 175)** | **Female (n = 66)** | **p value** |
| Age, years | 67 [58 – 73] | 75 [71 – 79] | < 0.001 |
| Sex, male | 175 (100%) | 0 (0%) | N/A |
| Body Mass Index, kg/m^2^ | 27 [25 – 30] | 27 [24 –29] | 0.10 |
| Diabetes | 44 (25%) | 22 (33%) | 0.26 |
| Multivessel disease | 110 (72%) | 28 (58%) | 0.08 |
| Recent myocardial infarction | 29 (17%) | 10 (15%) | 0.85 |
| Left Ventricular Function | … | … | 0.66 |
| Poor, < 30% | 7 (4.0%) | 1 (1.5%) | … |
| Moderate, 30-50% | 30 (17%) | 13 (20%) | … |
| Good, > 50% | 138 (80%) | 52 (79%) | … |
| COPD | 17 (10%) | 9 (14%) | 0.36 |
| Extracardiac arteriopathy | 18 (10%) | 10 (15%) | 0.37 |
| Neurological dysfunction | 4 (2.3%) | 2 (3.0%) | 0.67 |
| Previous cardiac surgery | 7 (4.0%) | 4 (6.1%) | 0.50 |
| NYHA class | … | … | 0.008 |
| I | 134 (77%) | 37 (57%) | … |
| II | 26 (15%) | 23 (35%) | … |
| III | 13 (7.4%) | 5 (7.7%) | … |
| IV | 2 (1.1%) | 0 (0%) | … |
| Urgency | … | … | 0.39 |
| Elective | 106 (61%) | 44 (67%) | … |
| Urgent | 64 (37%) | 21 (32%) | … |
| Emergency | 5 (2.9%) | 1 (1.5%) | … |
| Salvage | 0 (0%) | 0 (0%) | … |
| EuroSCORE I, logistic | 2.5 [1.5 – 5.5] | 6.4 [4.7 – 11] | < 0.001 |
| EuroSCORE II | 1.1 [0.82 – 1.8] | 2.1 [1.4 – 3.8] | < 0.001 |
| Data are means ± SD or Medians [IQR] or numbers (proportions)  COPD = Chronic Obstructive Pulmonary Disease; NYHA = New York Health Association | | | |

Baseline differences with p < 0.10 were added as factor or covariate to a linear mixed model for ACSM and time (days after surgery). Time remained significant in all models. Sex remained significant in all models.

| **Table S4.2.** ACSM predictors of sex subgroup patients as determined with linear mixed model | | |
| --- | --- | --- |
| **Model with time (days) and 1 baseline characteristic (univariate) for effects on ACSM score** | | |
|  | **Effect and 95% Confidence Interval** | **P value** |
| Age, years | -0.02 [-0.03 to -0.00] | 0.02 |
| Body Mass Index, kg/m^2^ | 0.01 [-0.02 to 0.04] | 0.53 |
| Multivessel disease | 0.19 [-0.15 to 0.54] | 0.27 |
| NYHA class | -0.0 [- 0.22 to 0.22] | 0.99 |
| EuroSCORE I, logistic | -0.03 [-0.06 to -0.01] | 0.01 |
| EuroSCORE II | -0.03 [-0.08 to 0.02] | 0.27 |
| **Model with time (days), sex, and 1 baseline characteristic for effects on ACSM score** | | |
|  | **Effect and 95% Confidence Interval** | **P value** |
| Age, years | -0.00 [-0.02 to 0.01] | 0.23 |
| EuroSCORE I, logistic | -0.02 [-0.04 to 0.01] | 0.16 |
| **Final model with time (days) and sex for effects on ACSM score** | | |
|  | **Effect and 95% Confidence Interval** | **P value** |
| Intercept | 0.55 [0.25 to 0.85] | < 0.001 |
| Time, days after surgery | 0.86 [0.81 to 0.92] | < 0.001 |
| Sex, male | 0.67 [0.36 to 0.97] | < 0.001 |
| Point estimate determined with linear mixed model analysis. | | |

***File S5 –*** ***Characteristics of CABG subgroup and effects on ACSM and TCT scores***

| **Table S5.1.** Baseline characteristics of patients with myocardial revascularization | | | |
| --- | --- | --- | --- |
| **Variable** | **UCG (n = 21)** | **PMG (n = 142)** | **p value** |
| Age, years | 68 [56-73] | 70 [63-75] | 0.10 |
| Sex, male | 18 (86%) | 111 (78%) | 0.57 |
| Body Mass Index, kg/m^2^ | 29 ± 4.6 | 28 ± 4.2 | 0.63 |
| Diabetes | 7 (33%) | 46 (32%) | 0.90 |
| Multivessel disease | 17 (90%) | 121 (90%) | 1.00 |
| Recent myocardial infarction | 6 (29%) | 33 (23%) | 0.59 |
| Left Ventricular Function | … | … | 0.30 |
| Poor, < 30% | 2 (10%) | 6 (4.2%) | … |
| Moderate, 30-50% | 2 (10%) | 30 (21%) | … |
| Good, > 50% | 17 (81%) | 106 (75%) | … |
| COPD | 2 (10%) | 14 (10%) | 1.00 |
| Extracardiac arteriopathy | 1 (4.8%) | 21 (14.8%) | 0.31 |
| Neurological dysfunction | 0 (0%) | 5 (3.5%) | 1.00 |
| Previous cardiac surgery | 0 (0%) | 2 (1.4%) | 1.00 |
| NYHA class | … | … | 0.20 |
| I | 21 (100%) | 115 (82%) | … |
| II | 0 (0%) | 18 (13%) | … |
| III | 0 (0%) | 7 (5.0%) | … |
| IV | 0 (0%) | 1 (0.7%) | … |
| Urgency | … | … | 0.48 |
| Elective | 9 (43%) | 72 (51%) | … |
| Urgent | 12 (57%) | 65 (46%) | … |
| Emergency | 0 (0%) | 5 (3.5%) | … |
| Salvage | 0 (0%) | 0 (0%) | … |
| EuroSCORE I, logistic | 2.28 [1.51-4.29] | 2.89 [1.68-6.36] | 0.09 |
| EuroSCORE II | 1.06 [0.80-1.61] | 1.33 [0.98-2.1] | 0.09 |
| Data are means ± SD or Medians [IQR] or numbers (proportions)  CABG = Coronary Artery Bypass Grafting; COPD = Chronic Obstructive Pulmonary Disease; NYHA = New York Health Association; PMG = Poster Mobilization Group; UCG = Usual Care Group. | | | |

| **Table S5.2.** Periprocedural characteristics of patients with myocardial revascularization | | | |
| --- | --- | --- | --- |
| **Variable** | **UCG (n = 21)** | **PMG (n = 142)** | **p value** |
| **Type of surgery** | … | … | 0.53 |
| CABG, isolated | 17 (81%) | 117 (82%) |  |
| CABG + valve surgery | 2 (10%) | 19 (13%) |  |
| CABG + other surgery | 2 (10%) | 6 (4.2%) |  |
| Off-pump CABG, no cardiopulmonary bypass | 11 (52%) | 55 (39%) | 0.23 |
| Cardiopulmonary bypass time, min | 103 [67-137] | 104 [84-128] | 0.94 |
| Aortic crossclamp time, min | 60 [47-82] | 73 [54-88] | 0.37 |
| Data are means ± SD or Medians [IQR] or numbers (proportions)  CABG = Coronary Artery Bypass Grafting; PMG = Poster Mobilization Group; UCG = Usual Care Group. | | | |

| **Table S5.3.** Effect of mobilization poster on ACSM activity levels and TCT scores | | | |  |
| --- | --- | --- | --- | --- |
| **Variable** | **Increase of score by poster *** | **95% Confidence Interval** | **P value** | |
| **ACSM score** |  |  |  | |
| Overall (n=241) | 0.19 | -0.15 to 0.53 | 0.27 | |
| CABG (n=163) | 0.28 | -0.10 to 0.66 | 0.15 | |
| Discharged home (n=154) | 0.35 | -0.04 to 0.75 | 0.08 | |
| **TCT score** |  |  |  | |
| **Bed** |  |  |  | |
| Overall (n=220) | 0.13 | -0.04 to 0.27 | 0.06 | |
| CABG (n=150) | 0.18 | 0.02 to 0.33 | 0.02 | |
| Discharged home (n=146) | 0.19 | 0.04 to 0.34 | 0.02 | |
| **Chair** |  |  |  | |
| Overall (n=220) | 0.34 | 0.18 to 0.49 | < 0.001 | |
| CABG (n=150) | 0.33 | 0.16 to 0.51 | < 0.001 | |
| Discharged home (n=146) | 0.36 | 0.19 to 0.52 | < 0.001 | |
| **Toilet** |  |  |  | |
| Overall (n=220) | 0.34 | 0.13 to 0.55 | 0.002 | |
| CABG (n=150) | 0.34 | 0.10 to 0.58 | 0.006 | |
| Discharged home (n=146) | 0.39 | 0.15 to 0.62 | 0.001 | |
| **Corridor** |  |  |  | |
| Overall (n=220) | 0.30 | 0.09 to 0.51 | 0.005 | |
| CABG (n=150) | 0.27 | 0.04 to 0.51 | 0.006 | |
| Discharged home (n=146) | 0.39 | 0.16 to 0.63 | 0.001 | |
| **Home trainer** |  |  |  | |
| Overall (n=220) | 0.24 | 0.04 to 0.44 | 0.02 | |
| CABG (n=150) | 0.26 | 0.03 to 0.48 | 0.02 | |
| Discharged home (n=146) | 0.34 | 0.09 to 0.59 | 0.007 | |
| **Stairs** |  |  |  | |
| Overall (n=220) | -0.06 | -0.16 to 0.04 | 0.21 | |
| CABG (n=150) | -0.06 | -0.17 to 0.05 | 0.29 | |
| Discharged home (n=146) | -0.03 | -0.15 to 0.10 | 0.66 | |
| ^*^ Point estimate determined with linear mixed model analysis. | | | |  |

| **Table S5.4.** Postoperative mobilization characteristics as determined by ACSM and TCT scores | | | | |
| --- | --- | --- | --- | --- |
| **Variable** | **Overall**  **(n = 241)** | **UCG (n = 32)** | **PMG (n = 209)** | **P value** |
| ACSM score at surgical ward, POD1 | 1 [1 – 2] | 1 [1 – 1] | 1 [1 – 2] | 0.01 |
| ACSM score at discharge | 5 [4 – 6] | 5.5 [4 – 6] | 5 [4 – 6] | 0.28 |
| **TCT score at surgical ward, POD1 *** | | | | |
| Lying in bed | 1 [1 – 2] | 1 [1 – 2] | 1 [1 – 2] | 0.24 |
| Sitting in a chair | 2 [2 – 3] | 2 [2 – 2] | 2 [2 – 3] | 0.001 |
| Walking to toilet | 1 [1 – 2] | 1 [1 – 1] | 1 [1 – 2] | 0.013 |
| Walking on corridor | 1 [1 – 2] | 1 [1 – 1] | 1 [1 – 2] | 0.038 |
| Cycling on home trainer | 1 [1 – 1] | 1 [1 – 1] | 1 [1 – 1] | 0.37 |
| Walking the stairs | 1 [1 – 1] | 1 [1 – 1] | 1 [1 – 1] | 0.99 |
| **TCT score at discharge *** |  |  |  |  |
| Lying in bed | 3 [3 – 3] | 3 [3 – 3] | 3 [3 – 3] | 0.53 |
| Sitting in a chair | 4 [4 – 4] | 4 [3 – 4] | 4 [4 – 4] | 0.007 |
| Walking to toilet | 4 [3 – 4] | 3 [3 – 4] | 4 [3 – 4] | 0.006 |
| Walking on corridor | 4 [3 – 4] | 3 [3 – 4] | 4 [3 – 4] | 0.03 |
| Cycling on home trainer | 2 [1 – 3] | 2 [1 – 2] | 2 [1 – 3] | 0.06 |
| Walking the stairs | 2 [1 – 2] | 2 [1 – 2] | 2 [1 – 2] | 0.47 |
| * 28 patients from PMG had incomplete data-collection for one of more days  Data are Medians [IQR] or numbers (proportions)  ACSM = Activity Classification Guide for Inpatient Activities score from the American College for Sports Medicine; PMG = Poster mobilization group; POD= postoperative day after intensive care unit discharge; TCT = Thorax Centrum Twente score; UCG = Usual care group. | | | | |

**File S6 - Validation of TCT and ACSM score**

| **Table S6.** Validation of TCT and ACSM score | | | | | | |
| --- | --- | --- | --- | --- | --- | --- |
|  | **ACSM score** | | | | | |
| **TCT score** | **I** | **II** | **III** | **IV** | **V** | **VI** |
| **Bed** |  |  |  |  |  |  |
| Continuously in bed (1) | 125 | 20 | 20 | 3 | 1 | 0 |
| Except during meals (2) | 27 | 82 | 103 | 44 | 18 | 7 |
| Only after lunch / at night (3) | 1 | 8 | 74 | 113 | 107 | 157 |
| ICC, r | 0.73, 95% CI 0.69 to 0.76 | | | | | |
| Cohen kappa | 0.73 | | | | | |
|  | **I** | **II** | **III** | **IV** | **V** | **VI** |
| **Chair** |  |  |  |  |  |  |
| Never (1) | 13 | 0 | 0 | 0 | 0 | 0 |
| Max 30 min / day (2) | 125 | 49 | 26 | 5 | 2 | 1 |
| 2-3 hours / day (3) | 11 | 49 | 109 | 53 | 23 | 9 |
| Mainly in chair (4) | 4 | 12 | 62 | 102 | 101 | 154 |
| ICC, r | 0.74, 95% CI 0.68 to 0.78 | | | | | |
| Cohen kappa | 0.75 | | | | | |
|  | **I** | **II** | **III** | **IV** | **V** | **VI** |
| **Toilet** |  |  |  |  |  |  |
| Never / N.A. (1) | 125 | 29 | 6 | 3 | 0 | 0 |
| Toilet chair or with supervision (2) | 24 | 56 | 69 | 16 | 5 | 6 |
| Independent 1-2 times (3) | 3 | 19 | 92 | 67 | 51 | 46 |
| Frequently independent (4) | 1 | | | | | |
| ICC, r | 0.75, 95% CI 0.46 to 0.86 | | | | | |
| Cohen kappa | 0.82 | | | | | |
|  | **I** | **II** | **III** | **IV** | **V** | **VI** |
| **Corridor** |  |  |  |  |  |  |
| Never / N.A. (1) | 153 | 102 | 168 | 81 | 55 | 20 |
| Max 20m / < 2x per day (2) | 0 | 7 | 20 | 58 | 46 | 66 |
| > 20m / max 4x per day (3) | 0 | 1 | 8 | 18 | 17 | 48 |
| > 5x per day (4) | 0 | | | | | |
| ICC, r | 0.79, 95% CI 0.21 to 0.91 | | | | | |
| Cohen kappa | 0.88 | | | | | |
|  | **I** | **II** | **III** | **IV** | **V** | **VI** |
| **Home trainer** |  |  |  |  |  |  |
| Never / N.A. (1) | 153 | 102 | 168 | 81 | 55 | 20 |
| With supervision, max 1x (2) | 0 | 7 | 20 | 58 | 46 | 66 |
| Independent, 2x (3) | 0 | 1 | 8 | 18 | 17 | 48 |
| Independent, > 2x (4) | 0 | 0 | 1 | 3 | 8 | 30 |
| ICC, r | 0.38, 95% CI -0.20 to 0.67 | | | | | |
| Cohen kappa | 0.65 | | | | | |
|  | **I** | **II** | **III** | **IV** | **V** | **VI** |
| **Stairs** |  |  |  |  |  |  |
| Never / N.A. (1) | 153 | 107 | 191 | 129 | 78 | 40 |
| With supervision (2) | 0 | 3 | 4 | 30 | 45 | 105 |
| Independent (3) | 0 | 0 | 2 | 1 | 3 | 19 |
| ICC, r | 0.21, 95% CI -0.15 to 0.47 | | | | | |
| Cohen kappa | 0.47 | | | | | |
| ICC = Intra-class Correlation, two-way mixed, consistency, average-measures  Numbers are unique patient measurements (910 in total, patient days with measurements) | | | | | | |

**File S7. Milestone analysis of Moving is Improving! study**

| **Table S7** In-hospital mobilization milestones achieved with exercise program of this study | | | | |
| --- | --- | --- | --- | --- |
|  | **ACSM Milestone** | **UCG (n = 32)** | **PMG (n = 209)** | **P value** |
| POD1 | II | 7 (22) | 90 (43) | 0.02 |
| POD2 | III | 21 (66) | 150 (72) | 0.48 |
| POD3 | IV | 22 (71) | 110 (64) | 0.43 |
| POD4 | V | 16 (67) | 64 (53) | 0.22 |
|  | **TCT Milestone** | **UCG (n = 32)** | **PMG (n = 188) *** | **P value** |
| POD1 | Sitting in a chair | 28 (88) | 180 (96) | 0.06 |
| POD2 | Corridor walk | 29 (90) | 171 (91) | 0.95 |
| POD3 | Cycling on home trainer | 11 (36) | 76 (48) | 0.19 |
| POD4 | Walking the stairs | 18 (75) | 50 (45) | 0.01 |
| * 28 patients from PMG had incomplete data-collection for one of more days  Data are numbers (proportions) with proportions of patients admitted to the hospital on each postoperative day.  ACSM = Activity Classification Guide for Inpatient Activities score from the American College for Sports Medicine; PMG = Poster mobilization group; POD= postoperative day after intensive care unit discharge; TCT = Thorax Centrum Twente score; UCG = Usual care group. | | | | |

**References**

1. Whaley, M.H., et al., *ACSM's guidelines for exercise testing and prescription*. 7th ed. 2006, Philadelphia, Pa.: Lippincott Williams & Wilkins. p. 168.
